# Supply of Fibrinogen Concentrate Across Queensland Health

**DOI:** 10.1111/1742-6723.70392

**Published:** 2026-11-09

**Authors:** Akmez Latona, Rohan Brennan, Praj Pothulu, John Casey, Alan Ho, Biswadev Mitra

**Affiliations:** ^1^ Emergency Department Ipswich Hospital Ipswich Queensland Australia; ^2^ LifeFlight Retrieval Medicine Toowoomba Queensland Australia; ^3^ School of Public Health and Preventive Medicine Monash University Melbourne Victoria Australia; ^4^ Faculty of Medicine University of Queensland Brisbane Queensland Australia; ^5^ System Procurement, Corporate Service Division Queensland Health Brisbane Queensland Australia; ^6^ Clinical Information Systems Support Unit Digital Health Branch, eHealth Brisbane Queensland Australia; ^7^ Australian Red Cross Lifeblood Melbourne Victoria Australia; ^8^ Queensland Cyber Infrastructure Foundation Brisbane Queensland Australia; ^9^ Emergency and Trauma Centre Alfred Care Group Bayside Health Melbourne Victoria Australia

## Abstract

**Objective:**

To describe fibrinogen concentrate (FC) supply across Queensland Health.

**Methods:**

Statewide FC supply from January 2016 to September 2026 was analysed by service type, geography, major trauma designation, viscoelastic haemostatic assay (VHA) availability and temporal trends.

**Results:**

Of 24,717 g FC supplied, 97.0% was distributed to 54 hospitals, 2.0% to retrieval services and 1.0% to ambulance services. Of hospital supply, 25.4% reached regional and 6.1% remote/very remote hospitals; 66.2% was supplied to major trauma services and 92.9% to VHA‐capable hospitals. Population‐adjusted FC supply increased from 8.19 to 59.2 g per 100,000 population between 2016 and 2025.

**Conclusion:**

FC supply has increased substantially across Queensland, extending to regional, remote and prehospital services. This highlights the value of statewide patient‐level utilisation data for haemovigilance.

## 1 Introduction

In Queensland, Australia, fibrinogen replacement is achieved using either cryoprecipitate or fibrinogen concentrate (FC), with FC incorporated into most critical bleeding algorithms [1]. FC use for acquired hypofibrinogenaemia is off‐label, with the approved indication limited to acute bleeding in congenital fibrinogen deficiency. With increasing uptake of FC in regional, remote and prehospital services across Queensland, characterising its statewide distribution is important to understand its penetration into clinical practice. Patient‐level FC utilisation data are fragmented across multiple information systems. However, centralised procurement through QH Central Pharmacy enables characterisation of FC supply across the statewide public health system. The aim of this study was to describe FC supply according to service type, geography and health service characteristics.

## 2 Methods

This was a retrospective descriptive study of FC supply across QH from 1 January 2016 to 30 September 2026 using QH Central Pharmacy data. Variables included supply date, service type and quantity supplied. FC supply was summarised by Hospital and Health Service (HHS), service type, Accessibility/Remoteness Index of Australia (ARIA+), major trauma service designation, viscoelastic haemostatic assay (VHA) availability and VHA type (Rotational thromboelastometry and Thromboelastography). Geographical and hospital capability classifications were restricted to hospitals, with ambulance and retrieval services reported separately. ARIA+ categories included urban, regional and remote/very remote [2]. Major trauma services were defined as Clinical Services Capability Framework Level 6 trauma services, with dedicated trauma teams and comprehensive on‐site specialist services [3]. VHA availability and type were classified using a previously published statewide assessment of Queensland Health hospitals [4].

Population‐adjusted FC supply was calculated per 100,000 population for complete calendar years, using Queensland population estimates from the Australian Bureau of Statistics [5]. Supply trends were described across the study period. Ethics exemption was granted by West Moreton Health (EX46‐26‐131424).

## 3 Results

Across 6692 supply transactions identified, a total of 24,717 g of FC was distributed across QH. Median statewide supply was 195 g/month (IQR: 114–257). Figure 1a illustrates the distribution across HHS. Gold Coast HHS received the greatest quantity of FC (7695 g), followed by Metro North HHS (4841 g).

**FIGURE 1:**
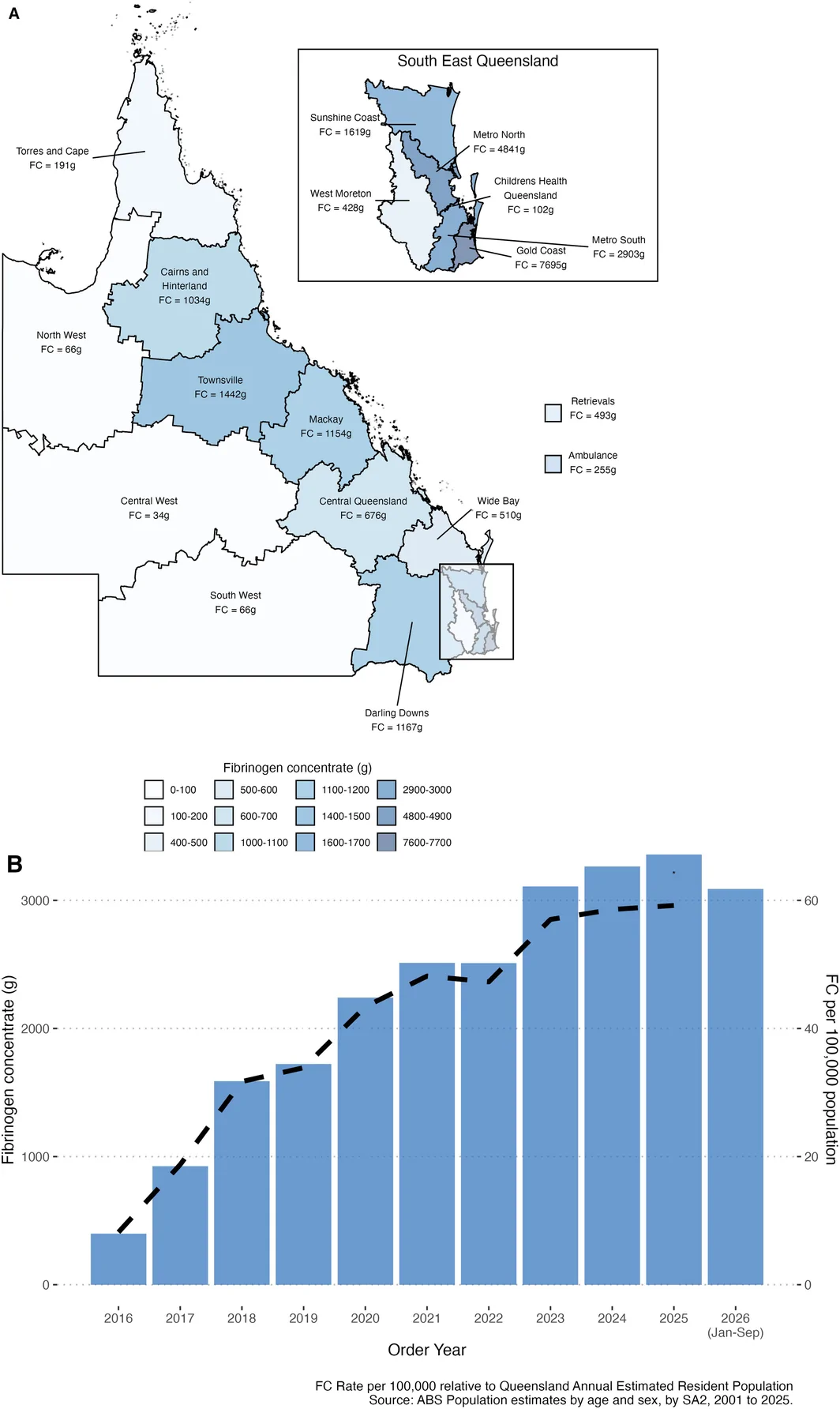
(a) FC supply by Hospital and Health Service. (b) Annual FC supply (bars) and population‐adjusted supply per 100,000 residents (dashed line) in Queensland, January 2016–September 2026.

Figure 1b shows FC supply over time. The overall FC supply increased steadily from 397 g in 2016 to 3357 g in 2025. The FC rate per 100,000 QLD population increased steadily as well from 8.19 g per 100,000 to 59.2 per 100,000.

Figure 2 and Supplementary Table 1 shows the distribution of FC by service type, geography and major trauma designation. Hospitals received 23,966 g (97.0%), retrieval services 493 g (2.0%) and the road ambulance service 255 g (1.0%). Hospital supply extended across 54 hospitals within 17 HHS.

**FIGURE 2:**
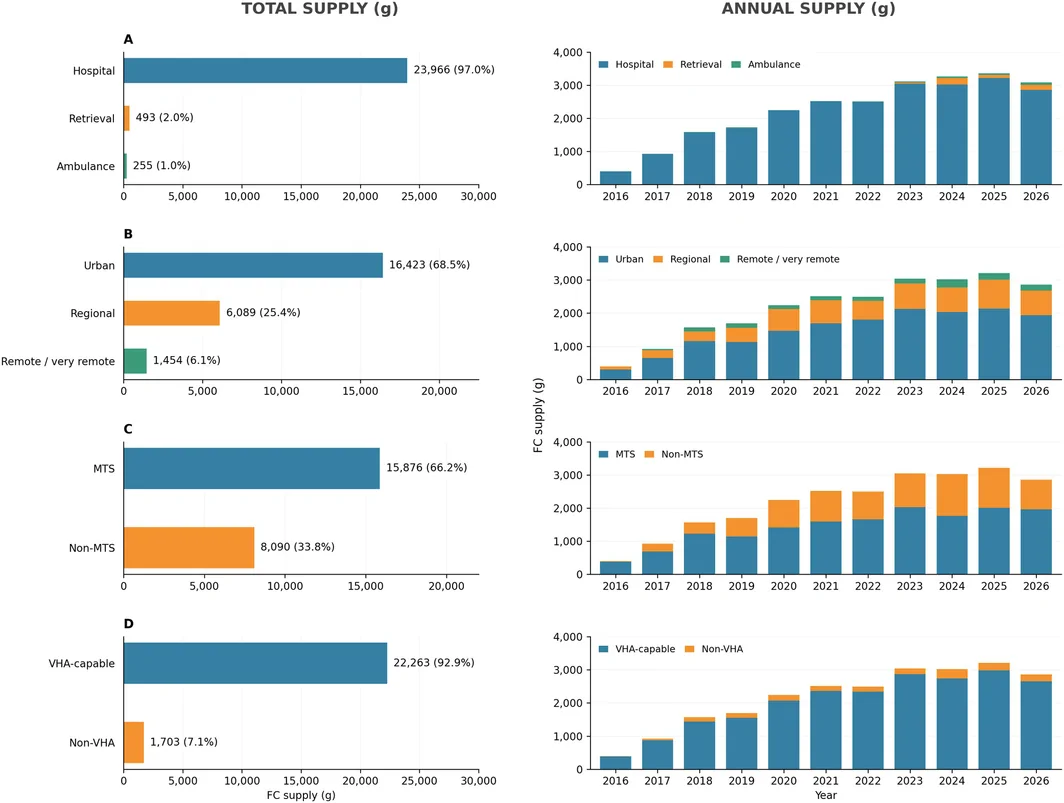
Total and annual fibrinogen concentrate supply across Queensland Health by service type, remoteness, major trauma service designation and VHA availability, January 2016–September 2026. (a) Service type. (b) Hospital geography (ARIA+). (c) Major trauma designation. (d) VHA availability.

Among hospitals, FC was predominantly supplied to operating theatres (7420 g; 31.0%), followed by blood bank (6245 g; 26.1%), emergency departments (4762 g; 19.9%) and intensive care units (3708 g; 15.5%). The remaining 1831 g (7.6%) were supplied to other clinical areas.

By hospital geography, 16,423 g (68.5%) was supplied to urban, 6089 g (25.4%) to regional and 1454 g (6.1%) to remote/very remote hospitals. Major trauma services received 15,876 g (66.2%) while VHA‐capable facilities received 22,263 g (92.9%).

Overall, 468 g (1.9%) of statewide FC was distributed for the FEISTY II study.

## 4 Discussion

FC supply across Queensland Health increased substantially between 2016 and 2025, after adjustment for population growth. Previous studies have reported increasing utilisation in tertiary hospitals in Queensland [6]. Our study extends this evidence to statewide supply, including regional, remote and prehospital services. Notably, almost one‐third of FC supply was distributed to regional and remote/very remote hospitals, demonstrating its availability in settings with more limited transfusion resources. This is consistent with incorporation of FC into major haemorrhage protocols across Queensland, including regional and remote hospitals [7]. Increasing uptake of VHA in regional hospitals may also have influenced FC supply outside metropolitan centres [4]. FC remains funded by individual hospitals rather than through National Blood Authority, with differing hospital funding models contributing to variation in access. National supply arrangements could support more equitable access.

Most FC supplied to hospitals (92.9%) was distributed to VHA‐capable facilities. This likely reflects both the greater patient volumes managed by these facilities and the incorporation of FC into VHA‐guided transfusion algorithms [1]. Fibrinogen replacement is commonly prioritised early in VHA‐guided algorithms, potentially contributing to increased demand for FC. The increase in FC supply over the study period coincided with the introduction of VHA algorithms and clinical trials investigating FC in trauma haemorrhage. FEISTY II trial accounted for only 1.9% of statewide FC supply recorded by Central Pharmacy [8]. This may underestimate trial‐related supply, as investigational stock could have been supplied external to standard Central Pharmacy arrangements. Nevertheless, FEISTY II may have increased clinician awareness and familiarity with FC. FC supplied to aeromedical retrieval services was largely attributable to the ROUGE trial, evaluating FC use in traumatic haemorrhage, although FC had been supplied to retrieval services prior to the trial's commencement in 2024 for both traumatic and non‐traumatic haemorrhage [9].

FC administration is not comprehensively captured within Queensland's centralised transfusion laboratory information system (AUSLAB), as supply predominantly occurs through pharmacy rather than blood bank systems. Only 26.1% of hospital FC supply was distributed to blood banks, highlighting that laboratory‐based records alone substantially under‐capture FC distribution. This limits patient‐level surveillance. Whether similar gaps exist across other Australian jurisdictions warrants investigation, particularly with the development of a National Transfusion Dataset underway. Ensuring capture of products distributed outside traditional transfusion laboratory systems will be important for comprehensive national transfusion surveillance. Our group will further investigate patient‐level FC administration, indications and outcomes using Queensland Health's integrated electronic Medical Record.

Our study has several limitations. Central Pharmacy data reflect FC supply rather than patient‐level administration, precluding assessment of clinical indication, administered dose, concurrent blood component use and patient outcomes. Supply may also reflect local stockholding and replenishment practices and should not be interpreted as clinical utilisation. Hospital service capabilities may influence FC supply, but the available data could not reliably attribute supply to individual specialties, such as cardiothoracic surgery and liver transplantation. The study was also limited to the QH public health system, excluding private hospitals.

## 5 Conclusion

FC supply has increased substantially across Queensland, extending beyond metropolitan hospitals to regional, remote, ambulance and aeromedical services. Its widespread distribution contrasts with the absence of statewide patient‐level utilisation data. Integrating FC administration data into existing transfusion information systems would support statewide haemovigilance.

## Funding

This work was supported by Emergency Medicine Foundation (EMJS‐490R44‐2025‐LATONA).

## Ethics Statement

West Moreton Health HREC (EX46‐26‐131424).

## Consent

The authors have nothing to report.

## Conflicts of Interest

The authors declare no conflicts of interest.

## Supporting information


**Supplementary Table 1.**: Distribution of FC supply by geography, service type, major trauma centre designation and availability of viscoelastic haemostatic assay testing.

## Data Availability

The data that support the findings of this study are available on request from the corresponding author. The data are not publicly available due to privacy or ethical restrictions.
