# Supplementary material for: Supply of Fibrinogen Concentrate Across Queensland Health
Source: Emerg Med Australas. 2026 Nov 9;38(6):e70392. doi: 10.1111/1742-6723.70392 (PMC13747690; doi:10.1111/1742-6723.70392)
Supplement: Supplementary file 1 [file EMM-38-0-s001.docx]

Supplementary Table 1: Distribution of FC supply by geography, service type, major trauma centre designation, and availability of viscoelastic haemostatic assay testing.

| **FC Supply g (%)** | **2016-2026** | **2016** | **2017** | **2018** | **2019** | **2020** | **2021** | **2022** | **2023** | **2024** | **2025** | **2026**  **(Jan-Sep)** |
| --- | --- | --- | --- | --- | --- | --- | --- | --- | --- | --- | --- | --- |
| **Total FC supply** | 24,717 | 397 | 924 | 1,588 | 1,724 | 2,242 | 2,513 | 2,511 | 3,108 | 3,264 | 3,357 | 3,089 |
| **Service Type** | | | | | | | | | | | | |
| Hospital | 23,966 (97.0) | 397 (100.0) | 924 (100.0) | 1,569 (98.8) | 1,696 (98.4) | 2,242 (100.0) | 2,513 (100.0) | 2,495 (99.4) | 3,041 (97.8) | 3,021 (92.6) | 3,210 (95.6) | 2,858 (92.6) |
| Retrievals | 493 (2.0) | 0 (0.0) | 0 (0.0) | 0 (0.0) | 0 (0.0) | 0 (0.0) | 0 (0.0) | 0 (0.0) | 42 (1.4) | 198 (6.1) | 100 (3.0) | 153 (5.0) |
| Ambulance | 255 (1.0) | 0 (0.0) | 0 (0.0) | 19 (1.2) | 28 (1.6) | 0 (0.0) | 0 (0.0) | 16 (0.6) | 25 (0.8) | 45 (1.4) | 47 (1.4) | 75 (2.4) |
| **Hospital Remoteness** | | | | | | | | | | | | |
| Urban | 16,423 (68.5) | 298 (75.1) | 648 (70.1) | 1,159 (73.9) | 1,128 (66.5) | 1,467 (65.4) | 1,691 (67.3%) | 1,804 (72.3) | 2,122 (69.8) | 2,036 (67.4) | 2,136 (66.5) | 1,934 (67.7) |
| Regional | 6,089 (25.4) | 92 (23.2) | 232 (25.1) | 288 (18.4) | 431 (25.4) | 656 (29.3) | 696 (27.7) | 564 (22.6) | 773 (25.4) | 738 (24.4) | 875 (27.3) | 744 (26.0) |
| Remote/Very Remote | 1,454 (6.1) | 7 (1.8) | 44 (4.8) | 122 (7.8) | 137 (8.1) | 119 (5.3) | 126 (5.0%) | 127 (5.1) | 146 (4.8) | 247 (8.2) | 199 (6.2) | 180 (6.3) |
| **Trauma Centre Designation** | | | | | | | | | | | | |
| MTS | 15,876 (66.2) | 375 (94.5) | 688 (74.5) | 1,227 (78.2) | 1,146 (67.6) | 1,416 (63.2) | 1,597 (63.5) | 1,659 (66.5) | 2,030 (66.8) | 1,768 (58.5) | 2,013 (62.7) | 1,957 (68.5) |
| Non-MTS | 8,090 (33.8) | 22 (5.5) | 236 (25.5) | 342 (21.8) | 550 (32.4) | 826 (36.8) | 916 (36.5) | 836 (33.5) | 1,011 (33.2) | 1,253 (41.5) | 1,197 (37.3) | 901 (31.5) |
| **VHA Availability** | | | | | | | | | | | | |
| VHA | 22,263 (92.9) | 385 (97.0) | 873 (94.5) | 1,443 (92.0) | 1,550 (91.4) | 2,066 (92.1) | 2,365 (94.1) | 2,342 (93.9) | 2,868 (94.3) | 2,736 (90.6) | 2,981 (92.9) | 2,654 (92.9) |
| Non-VHA | 1,703 (7.1) | 12 (3.0) | 51 (5.5) | 126 (8.0) | 146 (8.6) | 176 (7.9) | 148 (5.9) | 153 (6.1) | 173 (5.7) | 285 (9.4) | 229 (7.1) | 204 (7.1) |
| **VHA Type** | | | | | | | | | | | | |
| ROTEM | 20,398 (91.6) | 385 (100.0) | 835 (95.6) | 1,407 (97.5) | 1,504 (97.0) | 1,884 (91.2) | 2,164 (91.5) | 2,098 (89.6) | 2,618 (91.3) | 2,366 (86.5) | 2,656 (89.1) | 2,481 (93.5) |
| TEG | 1,865 (8.4) | 0 (0.0) | 38 (4.4) | 36 (2.5) | 46 (3.0) | 182 (8.8) | 201 (8.5) | 244 (10.4) | 250 (8.7) | 370 (13.5) | 325 (10.9) | 173 (6.5) |

Values are presented as grams (percentage). Percentages are calculated using the following denominators: total FC supply for Service Type; total hospital FC supply for Hospital Remoteness, Trauma Centre Designation and VHA Availability; and total FC supply to VHA-capable hospitals for VHA Type.Abbreviations: MTS: Major Trauma Service, VHA: Viscoelastic Haemostatic Assay, ROTEM: Rotational Thromboelastometry, TEG: Thromboelastography. 2026 data are from January to September.
